# Optimal excitation and emission wavelengths to analyze amino acids and optimize neurotransmitters quantification using precolumn OPA-derivatization by HPLC

**DOI:** 10.1007/s00726-015-1925-1

**Published:** 2015-02-18

**Authors:** J. Perucho, R. Gonzalo-Gobernado, E. Bazan, M. J. Casarejos, A. Jiménez-Escrig, M. J. Asensio, A. S. Herranz

**Affiliations:** 1 Neuropharmacology Laboratory, Neurobiology-Research Service, Hospital Universitario Ramón y Cajal, IRYCIS, Carretera de Colmenar, km 9,1, 28034 Madrid, Spain; 2 CIBERNED, Madrid, Spain; 3 Neurobiology Laboratory, Neurobiology-Research Service, Hospital Universitario Ramón y Cajal, IRYCIS, Carretera de Colmenar, km 9,1, 28034 Madrid, Spain

**Keywords:** Amino acids, HPLC, OPA, Excitation wavelength, Emission wavelength, Neurotransmitters

## Abstract

We describe an analytical methodology to obtain high sensitivity and better resolution through the study of fluorometric excitation (*λ*_ex_) and emission (*λ*_em_) spectrum wavelengths of OPA–amino acids. The spectrum emission study revealed a maximum signal peak at 450 nm for aspartate and glutamine. For glycine, taurine, and GABA, the maximum signal peak was at 448 and for glutamate at 452 nm. The remaining amino acids analyzed showed a maximum emission around 450 nm. The best signal obtained within the spectrum excitation experiments was using 229- to 450-nm *λ*_ex_–*λ*_em_. The drawbacks observed at these wavelengths were a baseline drift and negative peaks occurrence. Thus, the excitation wavelength of 240 nm was chosen (240- to 450-nm *λ*_ex_–*λ*_em_) as a compromise between a very good signal response and a baseline stability to resolve the 18 amino acids studied. Furthermore, this protocol was properly validated. On the other hand, the elution gradient program used for neuroactive amino acids (aspartate, glutamate, glycine, taurine and GABA) showed separation to the baseline, in a 15-min run in all of them. Other amino acids, up to 18, also exhibited a very good separation in a 25-min run. In conclusion, we propose the use of 240- to 450-nm *λ*_ex_–*λ*_em_ wavelengths, in OPA–amino acids analysis, as the most suitable protocol to obtain the best signal response, maintaining an optimum chromatographic resolution.

**Electronic supplementary material:**

The online version of this article (doi:10.1007/s00726-015-1925-1) contains supplementary material, which is available to authorized users.

## Introduction

The study of amino acid composition in samples of biological fluids, tissues homogenates or even from microdialysis samples is crucial to understand the homeostatic deficits of many diseases. The chromatographic separation of amino acids through HPLC columns has been a classic technique used since 1950 and has been studied and improved for decades to obtain better separation and resolution (Dai et al. 2014; Crescentini and Stocchi 1984; Stein and Brink 1981; Moore et al. 1958). In addition, numerous methods have been developed to increase the detection levels (Burriel Marti et al. 2000; Hancock 1984; Kubickova et al. 2011; Ersser and Davey 1991; Davey and Ersser 1990) and many authors continue using the HPLC methodologies to resolve amino acids in multiple mixture analysis (Molnar-Perl 2003; Haynes et al. 1991; Koros et al. 2007; Perry et al. 2009b). However, it is known that the pressure-driven hydrodynamic flow occurs independently of the column, making retention times very stable but with longer run times. Because of this issue, the reduction of the elution time has been a goal expressed by several authors (Devall et al. 2007; Oreiro-Garcia et al. 2005; Silva et al. 2007).

A common technique to analyze amino acids is to combine them with a specific molecule which can be easily detected by a fluorescence detector, separated by a reverse phase column in their specific retention times. Since its description in 1971 (Roth 1971), the use of OPA, as a fluorogenic agent for precolumn amino acids derivatization, has been widely accepted (Hanczko et al. 2007; Molnar-Perl 2001; Jones and Gilligan 1983). Almost every author describes a particular setting of excitation and emission wavelengths for the fluorescence monitoring of the thio-substituted isoindoles. A wide range of excitation wavelengths are reported: from 200 to 360 nm (*λ*_ex_) and from 420 to 455 nm for emission (*λ*_em_) (Molnar-Perl 2011; del Olmo et al. 2003; Tan et al. 2011; Ersser and Davey 1991). To our knowledge, the relationship between the *λ*_ex_ and *λ*_em_ wavelength and the intensity of fluorescence signal has not been described in any article.

The use of a JASCO^®^ fluorometer detector, with emission wavelength scan capabilities, has opened the possibility of determining the optimal excitation and emission wavelengths (*λ*_ex_–*λ*_em_) for OPA–amino acids. In this article we report the optimal excitation and emission wavelengths for 18 amino acids. In addition, the specifications of two elution programs which optimize the resolution and shorten the analysis time, and a validation study are also provided.

## Materials and methods

### Materials

Methanol HPLC grade was from Merck and sodium acetate was from Sigma-Aldrich. Ultrapure water and solvents were filtered through 0.20 µM filters from Millipore (Bedford, MA, USA). The amino acids’ external standard solution was a commercial mixture from Beckman (protein hydrolysate). Glutamine, GABA, taurine (Sigma-Aldrich) and tryptophan (Merck) were added individually. The final concentration of all amino acids present in the calibrated standard solution was 1.5 µM. Aliquots of standards were kept at −20 °C, being stable for at least 3 months.

### Derivatization procedure

The standards or samples were precolumn derivatized with 2-O-phthaldialdehyde (OPA) reagent solution. The derivatization reagent was: 32 mg OPA diluted in 800 µl of methanol, 7,140 μl of borate buffer 0.4 M (pH 9.5) and 60 μl of 3-mercaptopropionic acid (Sigma-Aldrich), freshly prepared every week and protected from light exposure. The derivatization reaction was performed with a programmable automatic injector (Gilson Mod. 231 XL, Middleton, USA). 10 μl of of OPA reagent was added to 20 µl of standard or sample. After 1 min of reaction, to stop it, 5 μl of 5 % acetic acid were added. 32 μl of the reaction mixture was injected into the analytical system. To avoid injecting bubbles 12 µl of the mixture was used as a flush volume, and 20 μl to totally fill the calibrated loop, remaining 3 µl in the mix tube. The precision of the injection method was measured as a coefficient of variation of area for ten injections, giving a CV <0.5 %.

### Chromatography system

The HPLC system consisted of two Jasco pumps (model PU-2080, Tokyo, Japan) and a fluorescence detector Jasco (model FP-2020, Tokyo, Japan) equipped with emission wavelength scan function. Moreover, different excitation/emission wavelengths can be selected. The fluorometer was equipped with a 16 µl flow cell. All injections were carried out with a Gilson 231 XL sampling injector (Middleton, US, 20 μl loop), equipped with a 720 key pad software that allows the automatization of the derivatization reaction. The thermostatic sample rack was maintained at 10 °C. Reversed-phase chromatography C18 columns “Ultrasphere ODS” (Beckman, USA) (150 × 4.6 mm, particle size 5 μm) were used.

### Solvents, elution programs and amino acid standards

Gradients were prepared with two degassed solvent mixtures. Solvent A was 0.05 M sodium acetate, pH 5.88, and methanol (95:5), and solvent B was methanol and double-distilled water (70:30). Degasification was performed by ultrasonic bath. According to the analysis requirements, two different elution programs were built up. A mixture of 18 amino acids was resolved using the gradient program 1 (“long”): initial conditions 25 % B, from 0.1 to 2.5 min, gradient step to 33 % B in 7 min, isocratic step at 60 % B for 3-min duration, gradient step to 80 % B in 9 min, isocratic step at 80 % B of 3 min, jump to 100 % B, isocratic step at 100 % B for 2 min and gradient step to 25 % B in 1 min; flow rate of 0.5 ml/min.

To achieve good separation of the neuroactive amino acids in a short elution period, a gradient program 2 (“short”) was carried out: initial conditions 25 % B, linear step to 25 % B from 0.1 to 2.5 min, gradient step to 33 % B in 7 min, gradient step at 60 % B for 3-min duration, jump to 100 % B, isocratic step at 100 % B for 4 min and gradient step to 25 % B in 1 min; flow rate of 0.5 ml/min.

**Fig. 1:**
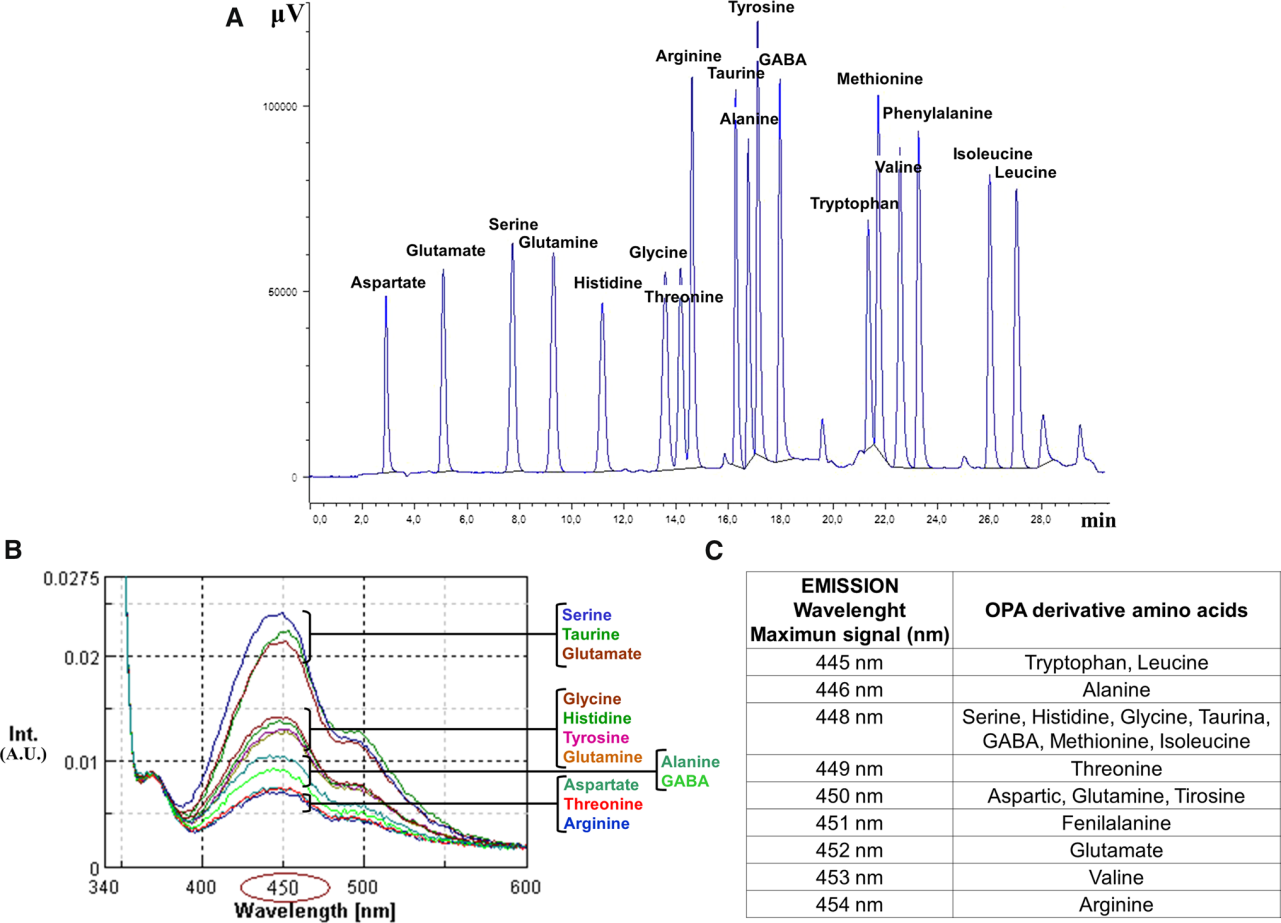
Representative chromatogram and emission wavelength scans of 18 OPA–amino acids. **a** Chromatogram of the 18 amino acids detected within the 25-min analysis at 240-nm *λ*_ex_ and 450-nm *λ*_em_. Each peak represents 17.14 pmol. **b** Overlapped emission scans of the 18 amino acids analyzed and signal intensity scaled in arbitrary units (*AU*). **c** Table summarizing the optimal emission wavelengths for the OPA–amino acids analyzed. Note that the 450-nm *λ*_em_ seems to be the optimal emission wavelength in order to perform the OPA–amino acids analysis

Amino acids were identified by their retention times, and their concentrations were calculated by comparison with calibrated amino acid external standard solution (1.5 µM). The amino acid pattern showed the next elution order: aspartate, glutamate, serine, glutamine, histidine, glycine, threonine, arginine, taurine, alanine, tyrosine, GABA, tryptophan, methionine, valine, phenylalanine, isoleucine and leucine (Fig. 1). This elution program was called “long run”. The “short run” covers in a 15-min analysis the first 12 amino acids described above. This protocol allows us to focus on the neuroactive amino acids: aspartate, glutamate, glutamine, glycine, taurine and GABA in a relatively short-timed analysis. In this case the elution order was: aspartate, glutamate, serine, glutamine, histidine, glycine, threonine, arginine, taurine, alanine, tyrosine and GABA.

The software used for the analysis and peak integration was ChromNAV, a software system from JASCO-HPLC analysis (Tokyo, Japan).

### Validation

#### Linearity

The calibration curves were obtained by plotting the amino acid peak areas versus eight different concentrations. These solutions with known concentrations were prepared by dilution of a stock solution of amino acids standard (8.57 pmol/µl) with double-distilled water to reach concentration ranges from 0.01675 to 8.57 pmol/µl for each amino acid. Each concentration was injected four times in the HPLC system. The correlation coefficient (*r* ^2^) for the linear equation *Y* = *mX* + *b* was calculated using linear regression least square method, where *Y* is the peak area and *X* denotes the concentration in pmol/µl of the amino acids.

The limits of detection (LOD) and quantification (LOQ) were calculated from the calibration curve of each amino acid according to the equations: LOD = 3.3 *σ*/*S* and LOQ = 10 *σ*/*S*, where *σ* is the standard error and *S* is the slope of the calibration curve (*n* = 4).

#### Accuracy and precision

To validate the assay procedure, the relative standard deviation (RSD %) and the relative error (RE %) of the mean measured concentration (*n* = 4) served as measures of accuracy and precision. The RE was expressed as the percentage deviation between nominal concentration and the observed concentration.

### Animals, ethics statement and samples preparation

All procedures used in this work were in accordance with the European Union Council Directive (2010/63/UE). The protocol was approved by the Committee on the Ethics of Animal Experiments of the Hospital Universitario Ramon y Cajal (animal facilities ES280790002001). The animals were housed three per cage in a temperature-controlled environment with 12 h light/dark cycles and access to food and water ad libitum, in an enriched environment with tissue paper and cardboard tubes. All efforts were made to minimize the number of animals used and their suffering.

The cortex and cerebellum were obtained from male Sprague–Dawley rats (180–220 g, *n* = 4). Rats were decapitated after 4 % isoflurane anesthesia and brains were rapidly dissected, frozen in dry ice and stored at −80 °C until analysis. The tissue was sonicated (VibraCell, level 2 for 30 s) in 8 volumes (*w*/*v*) of ice-cold 0.4 N perchloric acid (PCA) and then centrifuged for 20 min at 11,000*g* and 4 °C. The supernatants obtained from the deproteinized samples were diluted 1/750 in double-distilled water and then 20 µl was used for the derivatization procedure. Thus, the rat brain samples were neutralized to carry on the derivatization reaction in an appropriate pH range (9–9.5).

### Data analysis

Results are expressed as mean ± SEM of (*n*) independent samples. Statistical analyses were performed using Student’s *t* test or one-way ANOVA followed by the Newman–Keuls multiple comparison tests. Differences were considered significant when *P* ≤ 0.05. The analyses were performed using Graph Pad Prism software.

## Results

### Elution programs

A large number of studies have demonstrated that the amino acids mixtures derivatized with OPA can be resolved in a relatively short period of time by HPLC, using reverse phase C-18 columns and a elution gradient (Cereser et al. 2001; Hanczko et al. 2007; Molnar-Perl 2011). This 25-min run elution program allows us to detect and quantify 18 amino acids (Fig. 1a). Most of the peaks were resolved at baseline. Furthermore, a very good separation of glycine, threonine and the triple-peak composed by methionine, tryptophan and valine, which are usually difficult to accomplish with other methods, were also resolved.

After the establishment of this “long” elution method, we developed a “short” one, bearing in mind the amino acids of interest in neurobiology: aspartate, glutamate, glutamine, glycine, taurine and GABA. This shorter elution program (15 min) covers the analysis of the 12 first amino acids described in the 25-min run method. The complete elution order was: aspartate, glutamate, serine, glutamine, histidine, glycine, threonine, arginine, taurine, alanine, tyrosine and GABA.

### Emission spectra study

**Fig. 2:**
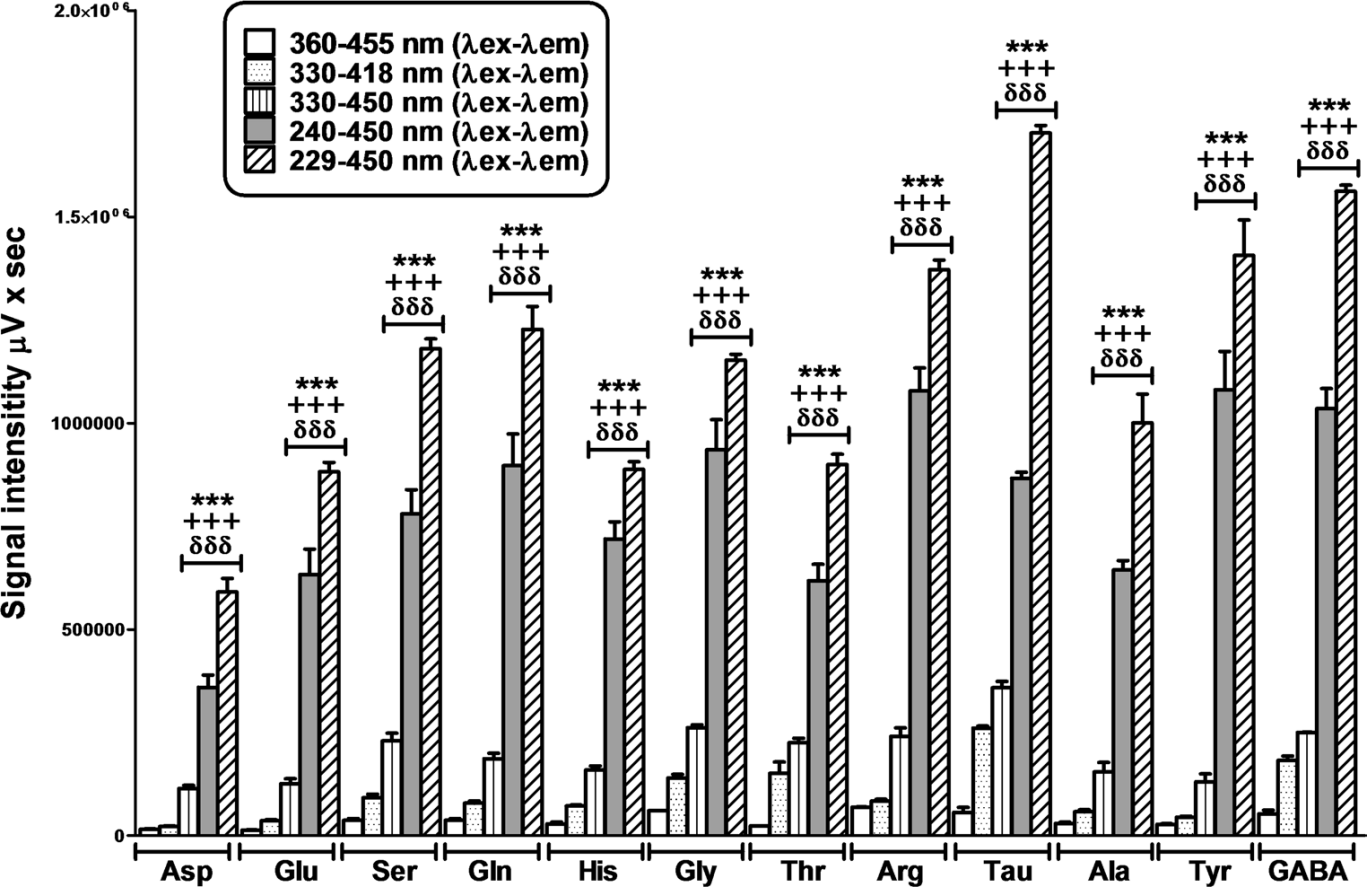
Analysis of five excitation and emission wavelength protocols. Initial conditions (365–455 *λ*_ex_–*λ*_em_), setup (330–418 *λ*_ex_–*λ*_em_), the new emission wavelength *λ*_em_ selected after the emission signal study (330–450 *λ*_ex_–*λ*_em_), the maximun signal obtained (229–450 *λ*_ex_–*λ*_em_) and the proposal by the authors (240–450 λ_ex_–λ_em_). There is a signal improvement in some of the amino acids using the initials conditions setup; however, this increase becomes general when the emission is set at 450 nm. The best signals were obtained with 240–450 and 229–450 λ_ex_–λ_em_. Data are expressed as the mean ± SEM (*n* = 5–8). The statistical analysis was performed using one-way ANOVA followed by Newman–Keuls multiple comparison test. ***P < 0.001, versus 360*λ*_ex_–455*λ*_em_; +++*P* < 0.001 versus 330*λ*_ex_–418*λ*_em_, ббб*P* < 0.001 versus 330*λ*_ex_–450*λ*_em_

The main goal of this work was to establish the optimum excitation and emission wavelengths for OPA–amino acids detection. First, a preliminary experiment was performed to compare the elution profile of the first 12 OPA–amino acids using two different excitation and emission conditions: our setup 360–455 nm (*λ*_ex_–*λ*_em_) (Mapelli et al. 2001; Rodriguez-Navarro et al. 2009; Herranz et al. 1984) and the 330–418 nm (*λ*_ex_–*λ*_em_) setup described by Jones and Gilligan (1983; Lindroth 1979) (data not shown). The 330- to 418-nm protocol versus 360–455 nm showed an improvement in signal detection of all amino acids studied, especially in glutamate, glycine, taurine and GABA (Fig. 2).

Because of these previous results, the emission spectra study was performed setting the excitation wavelength at 330 nm. The emission spectrum was framed in a window between 340 and 600 nm. The spectra data showed two peaks of maximum intensity for all OPA–amino acid studied, one of them near 450 nm and the other one with lower intensity around 500 nm (summarized in Fig. 1b, c). The optimal emission wavelengths (*λ*_em_) obtained for every amino acid were: aspartate 450 nm, glutamate 452 nm, serine 448 nm, glutamine 450 nm, histidine 448 nm, glycine 448 nm, threonine 449 nm, arginine 454 nm, taurine 448 nm, alanine 446 nm, tyrosine 450 nm, GABA 448 nm, methionine 448 nm, tryptophan 445 nm, valine 453 nm, phenylalanine 451 nm, isoleucine 448 nm and leucine 445 nm (Fig. 1c). To verify these optimal wavelength emission data, the experiments were repeated with different excitation wavelengths and similar results were obtained. These experiments indicated that all amino acids showed their maximum emission around 450 nm.

The signal intensity was compared among three methods: (1) our initial setup 360- to 455-nm *λ*_ex_–*λ*_em_ (Lerma et al. 1986), (2) 330–418 λ_ex_–λ_em_ from Jones et al. (1981) (Jones and Gilligan 1983) and (3) 330- to 450-nm, *λ*_ex_–*λ*_em_. This last method pointed out the largest increase in signal intensity in all amino acids versus the others two methods. Interestingly, the intensity of glycine, threonine, arginine, taurine and GABA peaks was increased between two and sixfold (Fig. 2). The results using the 450-nm emission protocol always showed a greater intensity signal at any excitation wavelength tested.

### Excitation wavelength study

The excitation studies were more complex due to the detector technical limitations. The study of excitation spectra (between 200 and 360 nm) was performed manually, adjusting the excitation wavelength every 5 nm and maintaining the emission wavelength at 450 nm. After this initial approach, more precise changes in wavelength selection were performed adjusting them to 1-nm variations to improve the accuracy of the excitation wavelength analysis.

**Fig. 3:**
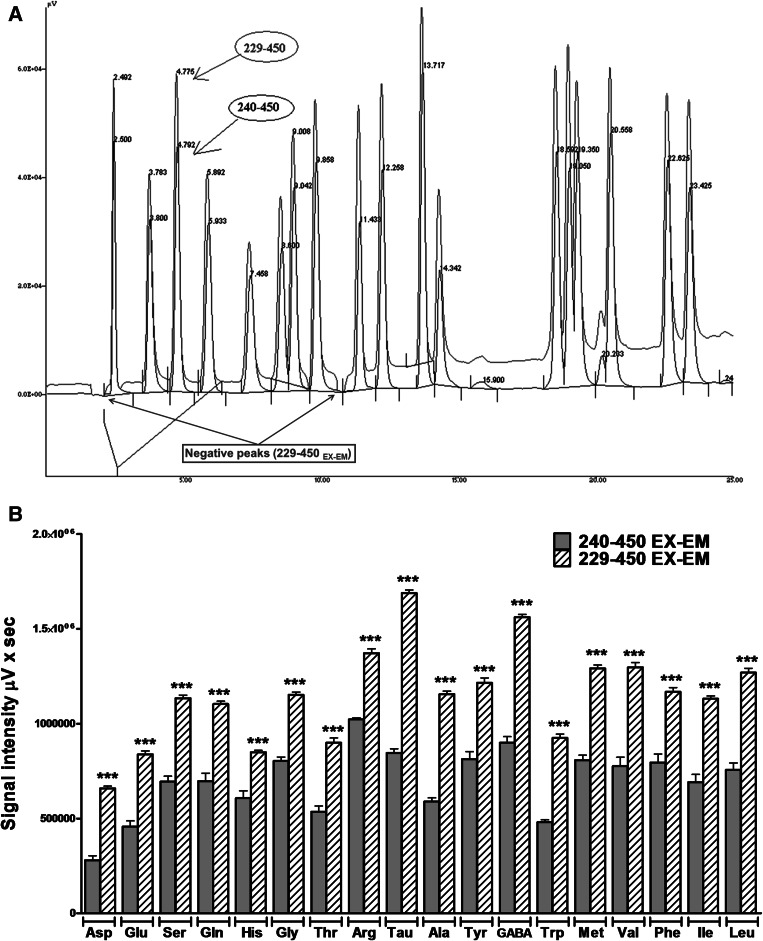
Comparison between the 229- and 240-nm wavelength excitation protocols maintaining the emission wavelength at 450 nm. **a** Examples of chromatograms analyzed at 229- to 450-nm (*blue*) and 240- to 450-nm (*black*) *λ*_ex_–*λ*_em_. Note how a baseline drift appears during the analysis using the 229- to 450-nm *λ*_ex_–*λ*_em_ protocol that it is not observed with the 240- to 450-nm *λ*_ex_–*λ*_em_ settings. Furthermore, the 240- to 450-nm *λ*_ex_–*λ*_em_ protocol showed a good fluorescence signal. **b** Statistical analysis of the 229- to 450- and 240- to 450-nm *λ*_ex_–*λ*_em_ setups. Note that the 229- to 450-nm *λ*_ex_–*λ*_em_ protocol provides an increased signal compared with the 240- to 450-nm *λ*_ex_–*λ*_em_ setup. Data are expressed as the mean ± SEM of three to six different experimental analyses of standards). Student’s *t* test analysis was performed. ***P < 0.001 versus 240*λ*_ex_–450*λ*_em_ (color figure online)

The excitation wavelength study revealed that the maximum intensity signal was detected at 229-nm *λ*_ex_. Using this excitation wavelength, the amino acids signal increased up to sixfold compared with the initial study conditions, 360- to 455-nm, *λ*_ex_–*λ*_em_ (Fig. 2). However, negative peaks appeared and a progressive increase in the baseline drift was observed (Fig. 1a SM). Using the 240-nm *λ*_ex_ protocol, these drawbacks were avoided (Fig. 3a, b). The experiments performed using the 240-nm wavelength excitation protocol showed peaks with a very good sensitivity, without baseline drift and negative peaks. Thus, the 240- to 450-nm *λ*_ex_–*λ*_em_ protocol was the compromise for an easy identification, detection and amino acids integration.

### Neuroactive amino acids analysis

**Fig. 4:**
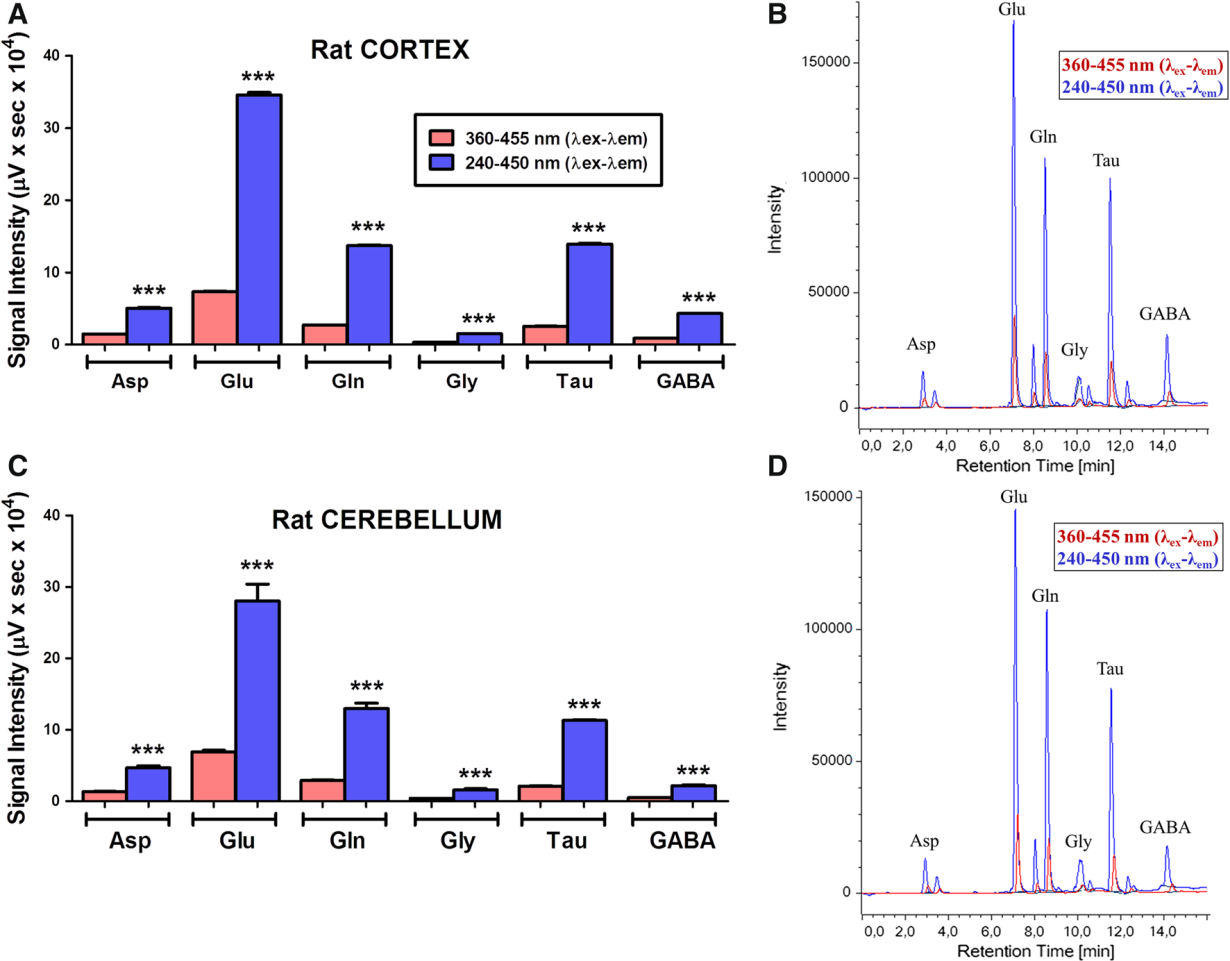
Analysis of neuroactive amino acids. Comparison of excitation–emission (*λ*_ex_–*λ*_em_) protocols. Statistical comparison of the 360- to 455- and 240- to 450-nm proposed protocol using samples of rat cortex **(a)** and cerebellum **(c)**. **b**, **d** Representative chromatograms of rat cortex and cerebellum samples, respectively. Data are expressed as the mean ± SEM of four independent samples. Statistical analysis was performed using the Student’s *t* test. ***P < 0.001 versus 365λ_ex_–455λ_em_

Considering the amino acids of interest in neurobiology (aspartate, glutamate, glutamine, glycine, taurine and GABA), the signal intensity produced by the amino acids present in homogenates samples of rat brain from cortex and cerebellum was compared, as shown in Fig. 4. The analysis and quantification of these neuroactive amino acids showed that the 229- to 450-nm *λ*_ex_–*λ*_em_ protocol produced a significant improvement in the signal compared with 240- to 450-nm *λ*_ex_–*λ*_em_ setup (Figs. 2, 3). However, baseline drift and negative peaks were also observed when excitation wavelength was set at 229-nm *λ*_ex_–*λ*_em_ (Figs. 3a, 1 SM). The 240- to 450-nm *λ*_ex_–*λ*_em_ settings showed significant improvement versus the 360- to 455-nm *λ*_ex_–*λ*_em_ setup in rat brain homogenates samples, offering also a stability desirable to perform biological sample analysis (Fig. 4a, c).

### Validation

The correlation coefficients of the standard curves (*r* ^2^ > 0.999) demonstrate the linearity of 240- to 450-nm *λ*_ex_–*λ*_em_ protocol, over the concentration range studied (Table 1). Taken all together, the coefficient results and LOD/LOQ data, it is plausible to indicate that 240- to 450-nm *λ*_ex_–*λ*_em_ protocol is suitable for quantifying the content of OPA–amino acids (Table 1). The RSD values for the three concentrations were <4 %, ranging from 0.57 to 3.94 % (Table 2). The relative error (RE %) of the mean measured concentration was also evaluated (Table 2).

**Table 1:** Characteristic parameters for the regression equations of the 240–450 *λ*_ex_–*λ*_em_ proposed protocol for determination of OPA–amino acids

Amino acid	Calibration range (pmol/μl)	LOD (pmol/μl)	LOQ (pmol/μl)	Equation	Correlation coefficient (*r* ^2^)
Asp	0.03347–8.57	0.0034	0.0103	*Y* = 27.230 *X* + 0.3365	0.99993
Glu	0.01675–8.57	0.0015	0.0046	*Y* = 45.9784 *X* + 0.3765	0.99995
Ser	0.06695–8.57	0.0007	0.0024	*Y* = 68.9961 *X* + 0.4255	0.99998
Gln	0.01675–8.57	0.0005	0.0014	*Y* = 82.7780 *X* + 0.3196	0.99998
His	0.06695–8.57	0.0066	0.0199	*Y* = 86.870 *X* − 0.9680	0.99991
Gly	0.03347–8.57	0.0012	0.0036	*Y* = 84.8501 *X* − 0.3534	0.99991
Thr	0.03347–8.57	0.0009	0.0028	*Y* = 53.8510 *X* − 0.2251	0.99998
Arg	0.01675–8.57	0.0005	0.0016	*Y* = 103.2197 *X* − 1.4574	0.99997
Tau	0.01675–8.57	0.0016	0.0049	*Y* = 92.9567 *X* − 0.7805	0.99979
Ala	0.01675–8.57	0.0010	0.0030	*Y* = 60.0545* X* + 1.5894	0.99997
Tyr	0.01675–8.57	0.0007	0.0022	*Y* = 82.1810 *X* + 0.3401	0.99997
GABA	0.01675–8.57	0.0024	0.0073	*Y* = 100.4494* X* − 1.9301	0.99945
Met	0.03347–8.57	0.0006	0.0018	*Y* = 81.3713* X* + 0.6964	0.99998
Trp	0.01675–8.57	0.0006	0.0019	*Y* = 74.8664 *X* + 0.0881	0.99998
Val	0.03347–8.57	0.0009	0.0027	*Y* = 79.5866 *X* + 2.6198	0.99996
Phe	0.01675–8.57	0.0004	0.0013	*Y* = 80.9832 *X* − 0.1642	0.99998
Ile	0.03347–8.57	0.0006	0.0020	*Y* = 69.8819 *X* − 0.0745	0.99998
Leu	0.01675–8.57	0.0005	0.0017	*Y* = 76.8850 *X* − 0.4095	0.99998

**Table 2:** Precision and accuracy validation of OPA–amino acids detection using the 240–450 *λ*_ex_–*λ*_em_ settings

Amino acids	Nominal conc. (pmol/μl)	Observed conc. (pmol/μl) ± SD	RSD (%)	Mean RE (%)
Aspartate	0.134	0.1353 ± 0.003	2.44	0.9701
	0.265	0.2605 ± 0.0045	1.74	−1.6981
	2.140	2.1,840 ± 0.0528	2.42	2.0561
Glutamate	0.134	0.1300 ± 0.003	2.26	−2.9851
	0.265	0.2585 ± 0.0059	2.29	−2.4528
	2.140	2.1,510 ± 0.0847	3.94	0.5140
Serine	0.134	0.1477 ± 0.003	2.23	10.2239
	0.265	0.2660 ± 0.0052	1.96	0.3774
	2.140	2.123 ± 0.04759	2.24	−0.7944
Glutamine	0.134	0.1323 ± 0.002	1.56	−1.2687
	0.265	0.2599 ± 0.0015	0.57	−1.92
	2.140	2.137 ± 0.0433	2.03	−0.1402
Histidine	0.134	0.1285 ± 0.0017	1.35	−4.1045
	0.265	0.26 ± 0.0055	2.13	−1.8868
	2.140	2.114 ± 0.04581	2.17	−1.2150
Glycine	0.134	0.1397 ± 0.0021	1.47	4.2537
	0.265	0.2611 ± 0.0046	1.75	−1.4717
	2.140	2.072 ± 0.024	1.16	−3.1776
Threonine	0.134	0.1285 ± 0.0021	1.62	−4.1045
	0.265	0.2536 ± 0.0057	2.24	−4.3019
	2.140	2.134 ± 0.06262	2.93	−0.2804
Arginine	0.134	0.1240 ± 0.0016	1.32	−7.4627
	0.265	0.2543 ± 0.0023	0.90	−4.0377
	2.140	2.128 ± 0.03418	1.61	−0.5607
Taurine	0.134	0.1370 ± 0.0014	1.03	2.2388
	0.265	0.2660 ± 0.0025	0.93	0.3741
	2.140	2.069 ± 0.04935	2.39	−3.3178
Alanine	0.134	0.1463 ± 0.0031	2.11	9.1791
	0.265	0.2701 ± 0.0042	1.56	1.92
	2.140	2.091 ± 0.04838	2.31	−2.2897
Tyrosine	0.134	0.1348 ± 0.0017	1.27	0.5970
	0.265	0.2620 ± 0.0022	0.86	−1.1321
	2.140	2.153 ± 0.06031	2.8	0.6075
GABA	0.134	0.1390 ± 0.0032	2.28	3.7313
	0.265	0.2638 ± 0.0033	1.24	−0.4528
	2.140	2.095 ± 0.04549	2.17	−2.1028
Methionine	0.134	0.1340 ± 0.0018	1.36	0
	0.265	0.2620 ± 0.0023	0.86	−1.1321
	2.140	2.139 ± 0.03962	1.85	−0.0467
Tryptophan	0.134	0.1353 ± 0.0039	2.86	0.9701
	0.265	0.2600 ± 0.0018	0.71	−1.8868
	2.140	2.107 ± 0.05028	2.39	−1.5421
Valine	0.134	0.1497 ± 0.0041	2.27	11.7064
	0.265	0.2711 ± 0.0046	1.71	2.3019
	2.140	2.106 ± 0.05925	2.81	−1.5888
Phenylalanine	0.134	0.1348 ± 0.0041	3.05	0.5970
	0.265	0.2623 ± 0.0018	0.70	−1.0189
	2.140	2.165 ± 0.05617	2.59	1.1682
Isoleucine	0.134	0.1345 ± 0.0044	3.30	0.3731
	0.265	0.2612 ± 0.005	1.91	−1.4340
	2.140	2.182 ± 0.05679	2.60	1.9626
Leucine	0.134	0.1348 ± 0.0033	2.45	0.5970
	0.265	0.2629 ± 0.0049	1.88	−0.7925
	2.140	2.129 ± 0.06657	3.13	−0.5140

## Discussion

In recent decades, primary amino acid analysis have led to many improvements; probably one of the most popular has been the HPLC precolumn derivatization with OPA/thiol (Kutlan et al. 2002). Nowadays, different protocols of OPA reagent components have been used, paying special attention, among other issues, to the blank value, composition, stability and lifetime of the reagents (Cereser et al. 2001; Kirschner and Green 2009; Molnar-Perl 2011). One of the essential points of chromatographic analysis using OPA is the appropriate choice of excitation (*λ*_ex_) and emission (*λ*_em_) wavelengths for detecting the maximum fluorescence signal. Here, we have studied different excitation and emission spectra to find out which combination of excitation/emission wavelengths produces the best fluorescence signal detection for derivatized amino acids with OPA/3-mercaptopropionic acid.

### Emission wavelength study

Our initial goal was to compare, using the “short” elution program mentioned in the methods section, our settings (360–455 *λ*_ex_–*λ*_em_) with other widely employed in classical chromatography studies (330–418 *λ*_ex_–*λ*_em_) (Mopper 1982; Jones and Gilligan 1983; Stein and Brink 1981; Jones et al. 1981; Lindroth 1979) (data not shown). The results indicated that the 330–418 *λ*_ex_–*λ*_em_ protocol was more efficient than our former setup. As a consequence of this study, we performed the emission wavelength analysis fixing at 330 nm the excitation wavelength as reference.

The results of the emission trial showed the maximum emission spectra response for all amino acids around 450 nm (Fig. 1b, c). In addition, the analysis of the spectrum results obtained in the “short” program revealed that amino acids can be classified into three groups depending on the intensity of their emission spectra: one with lower intensity (alanine, GABA, aspartate, threonine and arginine), a second group with medium-size intensity (glycine, histidine, tyrosine and glutamine) and finally a third group of high response (serine, taurine and glutamate) (Fig. 1b). Data obtained from the analysis of the emission spectra of the 18 amino acids confirm that the 450-nm *λ*_em_ wavelength shows the best sensitivity compared with other wavelengths used in several studies: 418-nm *λ*_em_ (Jones et al. 1981; Jones and Gilligan 1983) and 455-nm *λ*_em_ (Hancock 1984; Koros et al. 2007). Moreover, the individual emission spectra analysis indicated a biphasic behavior for each amino acid studied, with a maximum intensity peak near the 450-nm emission wavelength and a smaller peak near 500 nm (Fig. 1b). Furthermore, these numbers are close to the ones expected for maximum optimum fluorescence of the isoindoles, 337*λ*_ex_–454*λ*_em_, product of the derivatization with the OPA and amino acids (Hanczko et al. 2007).

### Excitation wavelength study

Since the results obtained in the emission wavelength analysis, the excitation spectra study was always performed using 450 nm as a preset emission wavelength. To reach a compromise between sensitivity and ease of chromatographic processing, experiments were performed starting at 200 nm, and increasing every 5 nm in a linear way, until 400-nm *λ*_ex_. Focusing on the range from 225 to 235 nm the maximum sensitivity was found at 229-nm *λ*_ex_, but the baseline was unstable and negatives peaks were found (Fig. 1 SM). The analysis of the excitation wavelengths from 240 to 250 nm showed a very good signal sensitivity that was not accompanied by negative peaks or baseline drifts. Interestingly, the analysis of amino acids with OPA derivatives showed a maximum peak response at 229-nm *λ*_ex_ and a drop in intensity up to values close to 330 nm. This second peak observed near 330-nm *λ*_ex_ was smaller than the one observed at *λ*_ex_ 229 nm. These data suggest the presence of possible “biphasic” behavior in the excitation spectra of the OPA–amino acids studied.

### Optimal excitation and emission wavelength

The use of the 229-nm excitation wavelength protocol, in conjunction with the specific data obtained from the spectra emission study, may be useful to perform a preferential detection of samples with low concentration of certain amino acids. With 229- to 450-nm *λ*_ex_–*λ*_em_ setup we found: (1) an increase in the sensitivity analysis in the 18 amino acids studied. This protocol also increases the detection of preferential amino acids in neurotransmission and neuromodulation such as glutamate, GABA, taurine, glycine, arginine and tyrosine. (2) The increased signal could lead to difficulties analyzing biological fluids, because contaminations could be magnified and cause problems in amino acid identification. (3) Moreover, there is an increase of the baseline drift and “negative peaks” at the beginning and medium part of the chromatogram analysis; these two issues could complicate the peaks integration and correct quantification.

To avoid these difficulties, we propose the use of the 240- to 450-nm *λ*_ex_–*λ*_em_ setup since our results clearly indicate that these analytical conditions also increase the detection levels of OPA–amino acids without losing chromatographic stability and resolution. Furthermore, the data presented in Tables 1 and 2 properly validate the results obtained using the 240- to 450-nm *λ*_ex_–*λ*_em_ setup.

### Neuroactive amino acid analysis

The amino acids glutamate, GABA, glycine, aspartate and taurine take part in the CNS physiology. Their role as excitatory or inhibitory neurotransmitters and neuromodulators (Perry et al. 2009a) is crucial to regulate the neuronal function. Our results show that the 240- to 450-nm *λ*_ex_–*λ*_em_ protocol increases the detection sensitivity of these neuroactive amino acids maintaining a chromatographic performance and reproducibility. This new protocol would be of interest in neuroactive amino acids analysis, focusing on single or simultaneous detection for applied neuroscience (Perry et al. 2009a; Piepponen and Skujins 2001). Our data shows a setup with a contrasted statistical improvement in sensitivity and reproducibility. This fact makes the use of the new wavelengths proposed interesting to apply to basic and clinical research neuroscience laboratories.

## Conclusion

The use of a fluorometer detector with variable excitation (*λ*_ex_) and emission (*λ*_em_) wavelength opens the possibility to determine the optimal *λ*_ex_–*λ*_em_ wavelengths in OPA derivatives. We have determined the optimal emission wavelengths (*λ*_em_) for each OPA–amino acid derivative for fluorometric detection: aspartate: 450 nm, glutamate 452 nm, serine 448 nm, glutamine 450 nm, histidine 448 nm, glycine 448 nm, threonine 449 nm, arginine 454 nm, taurine 448 nm, alanine 446 nm, tyrosine 450 nm, GABA: 448 nm, methionine 448 nm, tryptophan 445 nm, valine 453 nm, phenylalanine 451 nm, isoleucine 448 nm and leucine 445 nm. Data obtained in the study of the emission spectra of the 18 amino acids confirm that the wavelength of 450-nm *λ*_em_ to be the best for sensitivity. Our data also pointed out that the optimal excitation wavelength signal seems to be a compromise between ease baseline and sensitivity observed at 240-nm *λ*_ex_ and the maximum sensitivity obtained at 229-nm *λ*_ex_.

Summarizing, these results indicate that the use of 240- to 450-nm *λ*_ex_–*λ*_em_ as preset wavelengths in OPA–amino acids analysis is the more appropriate protocol to detect the best fluorescence signal without losing good chromatographic resolution and reproducibility. Furthermore, these new analytical conditions can be applied in the analysis of neuroactive amino acids that play a crucial role in the CNS physiology.

## Electronic supplementary material

Below is the link to the electronic supplementary material.

Fig. 1 SM Maximum signals and baseline instability observed with the 229–450 nm (*λ*_ex_–*λ*_em_) protocol. **a** Negative peaks and a progressive increase of the baseline drift were observed in the chromatograms. **b** Representative chromatograms obtained with the 229–450 nm (*red*), 240–450 nm (*blue*) and 330–450 nm (*black*) *λ*_ex_–*λ*_em_ protocols. The maximum signal was detected using the 229–450 nm (*red*) *λ*_ex_–*λ*_em_ protocol. Note the signal increase of neuroactive amino acids taurine and GABA. (TIFF 529 kb)

## Abbreviations

CNS: Central nervous system
GABA: Gamma aminobutyric acid
HPLC: High-performance liquid chromatography
OPA: O-phthaldialdehyde
PCA: Perchloric acid
*λ*_ex_: Excitation wavelength
*λ*_em_: Emission wavelength
