# Supplementary figures and images for: Optimal excitation and emission wavelengths to analyze amino acids and optimize neurotransmitters quantification using precolumn OPA-derivatization by HPLC
Source: Amino Acids. 2015 Feb 18;47(5):963–73. doi: 10.1007/s00726-015-1925-1 (PMC4412611; doi:10.1007/s00726-015-1925-1)

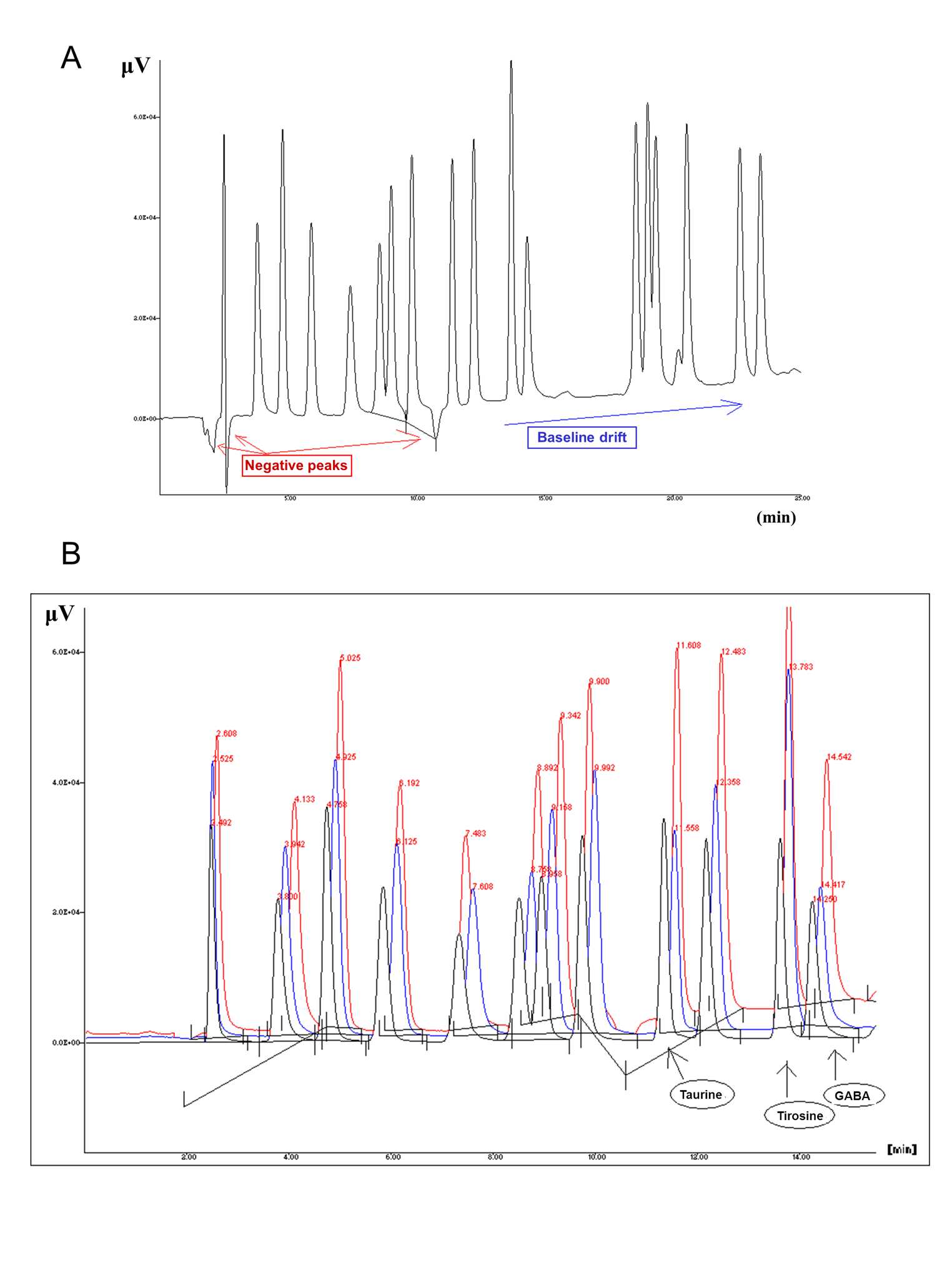

Supplement: Supplementary file 1 — Fig. 1 SM Maximum signals and baseline instability observed with the 229–450 nm (λ ex–λ em) protocol. a Negative peaks and a progressive increase of the baseline drift were observed in the chromatograms. b Representative chromatograms obtained with the 229–450 nm (red), 240–450 nm (blue) and 330–450 nm (black) λ ex–λ em protocols. The maximum signal was detected using the 229–450 nm (red) λ ex–λ em protocol. Note the signal increase of neuroactive amino acids taurine and GABA. (TIFF 529 kb) [file 726_2015_1925_MOESM1_ESM.tif]
